# Using selected behaviour modification practices to enhance reinforcement of reading abilities among dyslexic learners in Kenya

**DOI:** 10.4102/ajod.v10i0.707

**Published:** 2021-01-29

**Authors:** Pamela A. Ooko, Peter J.O. Aloka

**Affiliations:** 1 Faculty of Education, Teachers Service Commission of Kenya, Mombasa, Kenya; 2 Division of Studies in Education, Wits School of Education, University of the Witwatersrand, Johannesburg, South Africa

**Keywords:** selected, behaviour modification practices, enhancement, reinforcement, reading abilities, dyslexic learners, Kenya

## Abstract

**Background:**

Dyslexic learners have difficulties in accurate and fluent word recognition and poor spelling and decoding abilities.

**Objective:**

The present study investigated the use of selected behaviour modification practices to enhance reinforcement of reading abilities amongst dyslexic learners in primary schools in Kenya.

**Methods:**

The Solomon four research design was adopted. A sample size of 229 dyslexic learners in four selected schools was obtained using purposive sampling technique. The tools used were the Bangor Dyslexia Test and a short reading comprehension test. Internal validity of the constructs was tested using the Kaiser–Meyer–Oklin measure of sampling adequacy (KMO Index) and the Bartlett’s test of sphericity. The reliability of the questionnaires was ascertained using Cronbach’s alpha and internal consistencies of 0.673–0.807 were reported.

**Results:**

The findings reported a statistical significant difference between pre-test and post-test scores of the experiment group 1, *t* (48) = –15.059, *p* < 0.01, implying that a significant effect was found in the use of behaviour modification strategies in improving learner English language reading skills. The regression model explained 54.7% (*R*^2^ = 0.547) of the variability in the level of English language reading abilities amongst primary school learners with dyslexia.

**Conclusion:**

The study concludes that coaching behaviour modification practice had the highest influence on English language reading abilities as compared to prompting, shaping and modelling practices. The study recommended training of teachers on the use of behaviour modification practices to improve dyslexic learners’ reading ability.

## Introduction

Dyslexia is a learning disability with a neurobiological origin and is characterised by difficulties with accurate word recognition, poor spelling, and decoding abilities. These difficulties result from a deficit in the phonological component of language that is often unexpected in relation to other cognitive abilities and the provision of effective classroom instruction (Lyon, Shaywitz & Shaywitz 2003). The International Dyslexia Association (IDA 2017) described it as a specific learning difficulty in which learners have problems with poor spelling and decoding abilities. Dyslexia is a member of the family of learning disabilities; in fact, reading disability is by far the most common learning disability, affecting over 80% of those identified as learning disabled (Lerner 1989; Ooko, Aloka & Koweru 2019). On the contrary, Olagboyega (2008) defined dyslexia as a complex neurological condition which is constitutional in origin and may affect oral language skills, motor function, organisational skills and numeracy. According to Ondieki (2013), dyslexia is an impairment that interferes with fluency and accuracy when a person is reading and spelling words. The British Dyslexia Association (2013) defines dyslexia as a specific learning difficulty that interferes with the development of the ability to read and write. From the Kenyan context, the Kenya Dyslexia Organization (KDO) concludes that dyslexia is a neurologically based language deficiency which interferes with the acquisition and processing of language (IDA 2017). From the above definitions, dyslexia is a learning difficulty that includes reading, numeracy, motor and writing functioning amongst learners in schools. In the present study, the authors have adopted the definition of dyslexia by the KDO.

The primary symptoms of dyslexia are inaccurate or slow printed word recognition and poor spelling problems that, in turn, affect reading fluency, comprehension and written expression (Moats et al. 2010; Ooko, Aloka & Koweru 2019). The IDA (2017) reiterate that the symptoms of dyslexia include confusion with words that start with the letters b, p, d or g, memorising numbers and words, slow and laboured comprehension, and problems with arithmetical calculations and remembering days of the week in sequence or letters of the alphabet. Pirttimaa et al. (2015) reiterate that dyslexia is mainly caused by problems in phonological coding and the persistence of poor phonological skills. This idea is supported by Elbro and Scarborough (2004), who state that problems with phonological decoding and other challenges in phonological ability seem to be the core deficit in dyslexia. The prevalence of dyslexia in various countries has been reported, such as 1% of the Egyptian, 10% of the South African (Iwan 2013) and 20% of the Ugandan population. In Kenya, the prevalence of dyslexia is estimated to be about 10% (Symthe et al. 2004), as cited in Cheruiyot (2015). The dyslexic condition affects reading ability amongst learners in school, yet the ability to read is a necessary tool for everyone both in and out of school. Moreover, reading is important for the daily functioning of an individual. In schools, teachers use various skills to introduce reading amongst learners (Marima 2015). Boets and De Smedt (2010) reiterate that dyslexics are slower at grasping and less efficient when dealing with single-digit arithmetic or when identifying new words with sound-letter representations than typically developing students. They also find difficulty in analysing sounds of spoken words and putting them together with their respective letters as they read them. Students with dyslexia may harbour feelings of failure as a result of their dyslexic condition (Dyslexia Association in Australia 2014). Runo (2010) postulates that learners who score poorly in the wordlist and reading passage do not fare well academically in other subjects in primary schools.

The reviewed studies showed that students’ reading abilities attract attention of scholars in Kenya and in the global context. In Kenya and other parts of Africa, not many studies have been conducted on behaviour modification methods to improve dyslexics’ reading ability. Even though many studies have been carried out to strategise on how to remediate the reading ability of dyslexics, the problem is still unsolved. In addition, the relevant studies reviewed indicate that there are inconsistent findings about the extent to which behaviour modification strategies like prompting, shaping, coaching and modelling influence students’ reading behaviour. The purpose of this study was to determine the influence of selected behaviour modification practices on enhancement of reading ability amongst dyslexic learners in selected public primary schools in Kenya.

### Theoretical framework

According to the Dyslexia Association in Australia (2014), teachers need special training to enable them to accurately identify students who are struggling with reading and written language before attending to them using the special behaviour modification skills to improve their reading skills. This research was informed by Skinner’s theory of reinforcement. The theory was founded by Skinner (1957), as cited in Skinner (2011). Skinner’s reinforcement theory attempts to explain how behaviours of organisms are acquired and modified to improve a certain behaviour. Skinner believed positive reinforcement was more useful in modifying and reinforcing an already existing behaviour than when punishment was used. This theory was relevant to this study because it shows how reinforcement can be used with behaviour modification methods to improve reading abilities. From Skinner’s theoretical perspective, teachers can modify the reading ability of dyslexics by using behaviour modification practices such as coaching, shaping, prompting and modelling. Neitzel and Wolery (2010) define prompts as gestures, instructions, touches or things to make children correct responses independently. Mcleod (2015) defined shaping practice as a progressive way of achieving a desired behaviour by repeating a behaviour several times. Coaching can be defined as a means of transforming a research into a practice (Bowgren & Sever 2010). Cunningham and Allington (2010) define modelling as the technique of modifying reading ability by observing how teachers or models read the letters and their corresponding sound and then imitating the same.

### Literature review

Literature on various behavioural reinforcement practices and their effectiveness exists. For example, in Turkey, Backhaus, Jeske, Poinstingl and Koenig (2017) established that use of antecedent prompts was effective in transferring skills taught to young females. Similarly, Wong et al.’s (2016) study in the United States of America showed that the teachers’ conceptions of ‘Nature of Science’ improved significantly after two semesters of instruction. In New Zealand, Hayes (2010) found that prompting was effective in teaching autistic children. Allenger’s (2015) study in the United States of America showed that there were no significant differences between students’ word productions and sentence lengths with the teacher’s writing prompts. In India, Jeyasekaran’s (2014) study reported that there was a positive relationship between use of a visual, kinesthetic, auditory and tactile teaching method to improve the reading skill of children with dyslexia. Fonger and Malot’s (2018) study in the United States of America established that use of shaping was effective in teaching young children with autism (ASD). Mc Clurg and Morris (2014) in the United States of America reiterate that rewards are effective in motivating students to work hard.

In an experimental study with dyslexic learners, Ooko, Aloka and Koweru (2019) reported that there was a statistically significant positive relationship between shaping and reading abilities (*R*^2^ = 0.109). Majcharazak, Wagner and Yatez (2013) established that shaping is affected by three knowledge resources, which include adding domain knowledge to a Wiki. Heineke (2013) agreed that coaching can lead to improved teacher learning. Dusenbury et al. (2010) found that coaching was effective in enhancing the quality of implementation of drug abuse prevention programmes. In the United States of America, Joseph et al. (2015) revealed that a variety of strategies were used to teach self-questioning to students. In another study, Loh (2009) found that there was a significant positive relationship between teacher coaching and reading outcome. In the United Kingdom, Holliman et al. (2010) established that prosodic sensitivity is important in models of literacy development. Piper and Zulkowski (2015) found a statistically significant relationship between coaching and reading effect. In Kenya, Chacha’s (2018) study indicated that use of picture prompts was useful in the teaching of oral skills in prescholars. On the contrary, Ambrose and Cheong (2011) in Malaysia showed that the Clay Modeling Program does improve the reading behaviour of dyslexic children. Similarly, in Germany, Schukajilow, Krug and Rakoczy’s (2015) study reported that prompting students to find multiple solutions does not improve their performance directly. Maguire’s (2015) study in New Zealand showed that the video self-modelling intervention did not influence the participants’ reading habits.

According to Cassidy (2015), dyslexic learners can reach their full potential if remediated early as dyslexia is not a disability. In response to this, whole-word reading and phonetic methods have been employed to make a learner master reading in Kenya (Marima 2015). The phonetic method involves reading the sounds of letters and matching them to what each letter stands for. However, little has been realised on the efficacy of the phonetic method. According to Chitiyo and Wheeler (2009), behaviour can be modified by changing classroom routine and incorporating strategies such as shaping, modelling, coaching and reinforcement to improve behaviour. Dutta and Bhakta (2016) reiterate that behaviour modification is a way of using operant conditioning to replace undesirable behaviour with more desirable behaviour. The association between dyslexia and behaviour issues cannot be underscored, hence the need for behaviour modification to cater for such learners (Hulme &Snowling 2011). In Kenya, there are schools to cater for those learners with emotional and behaviour issues in approved schools, although the schools do not cater to the dyslexic learners (Muthee, Murugami &Tekle 2015).

It is estimated that about 10% of schoolgoing learners in Kenya could have dyslexia, although no official statistics are available for all regions in the country (Ooko, Aloka & Koweru 2019). Generally, there is lack of research in the area of dyslexic learners in Kenya and possible interventions are also not documented from the available research. One of the recent available studies by Ooko and Kaluyu (2016) found out that there is a significant weak positive relationship between writing dyslexia and academic performance amongst learners in upper primary public schools in Changamwe sub-county, Kenya. The study by Ooko and Kaluyu (2016) recommended a formulation of education policies catering to screening, teaching, learning, assessment and examination of dyslexic pupils in public primary schools in Kenya. Thus, the present study made follow-up to develop an intervention to enhance reading abilities amongst dyslexic learners in primary schools. The findings of the present study are significant because they would add to the body of literature and also be used by policymakers to provide early intervention programmes for pupils with dyslexia. The present study investigated the use of selected behaviour modification practices to enhance the reinforcement of reading abilities amongst dyslexic learners in primary schools in Kenya.

The research hypotheses of the study are stated as follows:

**H_o:1_** There are no significant differences in pre-test and post-test scores on English language reading ability between the dyslexic learners in experimental and control groups.

**H_o:2_** There is no significant effect of selected behaviour modification practices to enhance reinforcement of reading abilities among dyslexic learners.

## Research methods and design

### Study approach and design

The study was based on a quantitative research paradigm. The paradigm was adopted because it helped to make comparisons (Babbie 2010; Creswell 2014) of the four groups to ascertain the effects of the intervention of behaviour modification techniques. Specifically, the Solomon four research design was adopted. According to Solomon (1949), this is a randomised experimental design consisting of two treatments versus two control groups. The sample size was subdivided into four groups. In the first group (experimental 1) a pre-test was given to test the reading ability of the learners but there was no post-test. In the second group (control) a pre-test was given and a post-test to check if the intervention had an effect on their reading ability. In the third group (experimental 2) no pre-test was given; intervention was given and a post-test was given. In the fourth group (control) there was no pre-test or intervention but there was a post-test to check if they could read the words in the reading test effectively. This design was chosen for the study because it makes it possible to examine both the main effects of testing and the interaction of testing and treatment; thus it enabled to assess the presence of pre-test sensitisation (Fain 1999). A diagrammatic representation of the Solomon four research design is presented in Table 1.

**TABLE 1:** Diagrammatic representation of the Solomon four research design.

Group	Pre-test	Training	Post-test
Experimental group 1	Q1	X	T2 Q2
Control group 1	Q3	-	T2 Q4
Experimental group 2	-	X	T2 Q5
Control group 2	-	-	T2 Q6

*Source*: Adapted from research methods in education by Cohen, L., Manion, L., & Morrison, K., 2007, *Research Methods in Education (6th ed.)*, Routledge Falmer, London and New York

### Setting

The study was carried out in four public primary schools in Changamwe sub-county, Kenya. The selected schools had a high number of dyslexic learners and had not been performing well in the final examinations (Ministry of Education 2017). The researcher carried out the research in the sub-county as some cases of dyslexia had been identified. The selected primary schools were mainstream and inclusive in nature, and the teachers had no structured interventions on literacy. The Bangor Dyslexia Test was used to screen those confirmed to have dyslexic characteristics. The Hardin Simmons was used to rescreen learners identified with dyslexic characteristics.

### Study population and sampling strategy

The target population was 3267 learners and 54 English language teachers from seven schools. The researcher with the help of the 54 English language teachers selected a purposive sample of 229 dyslexic learners using the Hardin Simmons University (2014) Dyslexia Characteristics tool. The learners were in grades 5–8 and in age range from 10 to 15 years and consisted of both male and female learners. Purposive sampling is the deliberate choice of an informant because of the qualities the informant possesses. This sampling technique was adopted because it helped the researchers decide the individuals who can and are willing to provide information by virtue of knowledge or experience (Lewis & Sheppard 2006). The researcher further screened the learners using a Bangor Dyslexia Test (Miles 1997) to make sure those whose poor reading ability was caused by other factors were not included in the final sample.

### Intervention

The intervention on behaviour modification was developed on four practices, namely, prompting, shaping, coaching and modelling. Prompting was done by giving hints on letters or words where learners got stuck. For each word read correctly a tick was indicated in the score sheet. The learners were reinforced by use of praises and tokens when they performed the action correctly. For words which were prompted and they never managed to read correctly, a dot was used for marking on the scoresheet so that the following day they could be taken through the same process to read them. Shaping was done by progressively introducing words and sentences to the learners. Activities were picked from one part of the book *Dyslexia Workbook* by Morris (2012) daily and presented to the learners. The teacher then demonstrated to them how to read the words before they were asked to read them out on their own and observations were made on what they can read in a scoresheet. This was done by chunking the words into smaller portions and training the learners to read them out in smaller chunks. Where a learner had difficulty identifying the word from the list given, a ruler was used as a graphic organiser to help them locate the word in reference.

Coaching was done by the teacher acting as a coach and training the learners to read the words aloud. Every day activities were picked from one part of the book labelled *Dyslexia Workbook* by Morris (2012). The teacher then demonstrated to them how to read the words before they were asked to read them out on their own and observations made on what they could read in a scoresheet. Modelling was done by a teacher acting as a model to demonstrate how to read out the words by mapping out the sounds and the letters that they represented. Teachers carried out reading activities that were modelled to the learners. If a learner made a mistake in reading out the word aloud, the teacher corrected the learner by showing him or her how to read it out correctly and gave immediate feedback. Everyday activities were picked from one part of the book labelled *Dyslexia Workbook* by Morris (2012). The teacher then demonstrated to them how to read the words before they were asked to read them out on their own and observations made on what they could read in a scoresheet.

### Research tools

The Bangor Dyslexia Test was used to screen those confirmed to have dyslexic characteristics. The Hardin Simmons was used to rescreen learners identified with dyslexic characteristics. The Behaviour Modification Questionnaire had items on intervention for dyslexic learners, which included prompting, shaping, coaching and modelling. In addition, the researcher gave a short reading comprehension test to assess learners’ reading ability and a writing test to assess learners’ ability to organise ideas. External validity of the questionnaires was ensured by piloting the instruments in one non-sampled school using 20 learners and 4 teachers. The internal validity results of Bartlett’s test for sphericity were significant (*p* < 0.001, *p* = 0.000) and Kaiser–Meyer-hold Olkin indexes were 0.730, 0.685, 0.674 and 0.819 for all >0.6 for prompting, shaping, coaching and modelling subscales of the questionnaire, respectively. Reliability of the questionnaires was ensured by Cronbach’s alpha method and all the sub-scales met the required level of internal consistency of reliability with Cronbach’s alpha values ranging from 0.673 to 0.807.

### Data collection

Permission to carry out the research was first obtained from the National Council for Science, Technology and Innovation in Kenya. The instruments were pilot tested in one non-sampled school using 20 learners and 4 teachers. The researcher then sought consent from parents or guardians of the sampled dyslexic learners. The English teachers were trained for 1 month on behaviour modification practices and intervention practices. Learners who exhibited dyslexic characteristics were then screened by the researcher using the Bangor Dyslexia Test. The researcher subdivided the learners listed as dyslexics into four groups. The teachers helped in giving reading activities to the dyslexics to test the entry behaviour of the dyslexics for the pre-test scores. In one of the groups an intervention was given before the post-test. The trained teachers taught the dyslexic learners twice a week for 40 min a session for a period of 12 weeks using the interventions for the two experimental groups. After the intervention period, a post-test was given to the learners in two groups.

### Data analysis

Descriptive statistics such as mean, frequencies, percentages and standard deviations were used to analyse the data. In addition, inferential statistics such as *t*-test, Pearson’s product moment correlation and regression analyses were used to analyse data. Shapiro–Wilk’s test (S–W) was used to interpret the normality of the variables for small and medium samples up to *n* = 2000 (Creswell 2014). It was evident that all the other variables followed normal distribution given that there were no statistically significant differences noted in any of the variables with their corresponding normal scores. Therefore, Pearson’s product moment correlation and regression analysis were used in the analysis.

### Ethical consideration

This study was approved by the National Commission for Science Technology and Innovation in Kenya. The ethical clearance permit number is NACOSTI/P/19/5933/27531.

Consent was sought from the parents before screening of the learners was done. The parents then signed the letters of informed consent and returned them to the school before the learners participated in the study. The learners’ real names were not revealed to ensure anonymity, whilst those taking the Bangor Test were allowed to opt out voluntarily if they did not want to participate in the study. The interventions were carried out in classes to ensure confidentiality.

## Results on influence of behaviour modification practices on English language reading abilities

The study investigated the influence of selected behaviour modification practices on reading abilities amongst learners with dyslexia in public primary schools.

### Age distribution of the respondents

**FIGURE 1:**
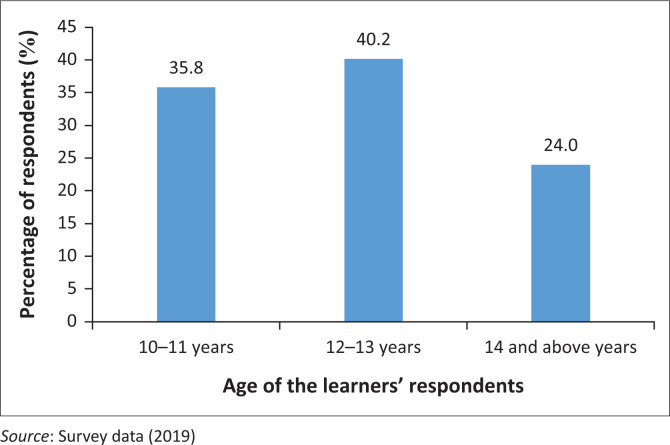
Age distribution of the respondents.

Information on the age distribution of respondents was sought and the results are presented in Figure 1.

The findings of the study show that the majority of the learners who took part in the survey were aged between 12 and 13 years; this was not surprising because although the study targeted learners from grades 5 to 8, most of the sampled learners were in grades 6 and 7. In Kenya, learners in these classes are mostly aged between 12 and 13 years. However, other age groups were also represented in the study.

### Differences in English language reading ability between the experimental and control groups

The study examined differences in pre-test and post-test scores on English language reading ability between the dyslexic learners in the experimental and control groups. To achieve this, a paired sample *t*-test was used to determine the difference in English language reading ability between the experimental and control groups. The different combinations of pre-tested and un-pre-tested groups with treatment and control groups allowed the researcher to ensure that confounding variables and extraneous factors did not influence the results on English language reading ability. Four groups of learners were considered and were labelled 1, 2, 3 and 4, respectively. Out of the four groups, group 1 and group 3, the experimental groups, were given treatment by training them on behaviour modification skills. On the contrary, group 2 and group 4 were not treated but only received the traditional teaching of English language reading. However, group 1 and group 2 were pre-tested before and post-tested after, whereas group 3 and group 4 were only post-tested. The results in Table 2 show the descriptive statistics analysis.

**TABLE 2:** Descriptive statistics of the scores of the four groups.

Group	*N*	Mean	Std. deviation	Std. error	95% Confidence interval for mean	
					Lower bound	Upper bound
**Pre-test scores**						
Group 1	49	24.90	9.599	1.371	22.14	27.66
Group 2	54	24.13	9.239	1.257	21.61	26.65
Group 3	0	-	-	-	-	-
Group 4	0	-	-	-	-	-
Total	103	24.50	9.373	0.924	22.66	26.33
**Post-test scores**						
Group 1	49	33.14	8.080	1.154	30.82	35.46
Group 2	54	25.30	11.843	1.612	22.06	28.53
Group 3	49	32.39	7.905	1.129	30.12	34.66
Group 4	52	26.35	11.383	1.578	23.18	29.52
Total	204	29.15	10.570	0.740	27.69	30.61

**FIGURE 2:**
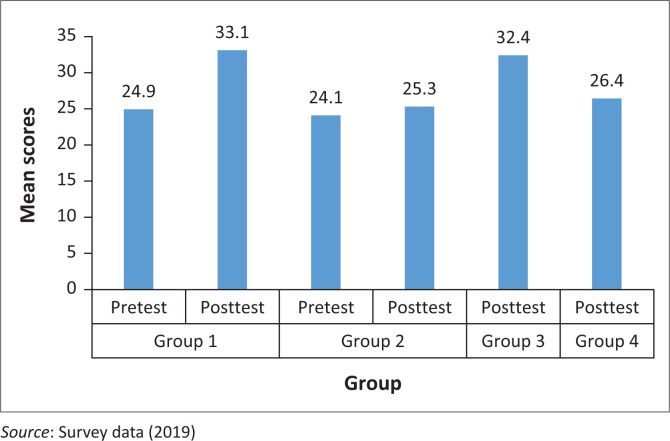
Mean scores in performance in English language reading.

From Table 2, it is evident that the highest mean score recorded was 33.1 (SD = 8.1) in the post-test reading by group 1 learners who received special treatment on behaviour modification strategies after a pre-testing. It was followed closely by the mean score of group 3 learners, who received treatment but were post-tested without pre-testing, at 32.4 (SD = 7.9) in a post-test examination. The least score recorded was pre-test results (*n* = 54, *M* = 24.13; SD = 9.2) for group 2 students, those who did not receive any special treatment on behaviour modification strategy. Figure 2 further shows the relative difference in mean scores of the various groups.

It is evident from Figure 2 that groups that received treatment reported relatively higher abilities in English language reading than their counterparts, who did not receive treatment. However, to investigate whether there is any statistically significant influence of behaviour modification strategies in English language reading abilities, three different steps involving the use of *t*-test were applied and findings were compared. Table 3 shows a comparison between the post-test scores attained by group 3 and group 4 learners.

**TABLE 3:** Paired samples test.

Pair	Paired differences					*t*	*df*	Sig.
	Mean	Std. deviation	Std. error mean	95% confidence interval distributions (CID)				
				Lower	Upper			
**Pair 1**								
Group 3 post-test – Group 4 post-test	5.979	12.1732	1.73903	2.4830	9.4761	3.438	48	0.001

*Source:* Survey data (2019)

The results in Table 3 show paired sample *t*-test investigating solution with the post-test only design with non-equivalent control groups. From the results, it can be concluded that there is a significant difference between the experimental group (group 3) and control group (group 4), (*t* (48) = 3.438; *p* = 0.001 < 0.05). Given that the difference is statistically significant at 5% level, it was concluded that the behaviour modification strategy is effective in improving English language reading abilities amongst the primary school learners. However, it is not known whether the existing difference in English language reading abilities is exclusively because of use of behaviour modification strategies or any other superseding variable that is not included in the survey. Therefore, the study further explored solution with the two control group design, as refinement over the finding, as shown in Table 4.

**TABLE 4:** Paired samples test: Solution with the two group control group design.

Pair	Paired differences			*t*	*df*	Sig.
	Mean	Std. deviation	Std. error mean			
**Pair 1**						
Group 1 pre-test – Group 1 post-test	−8.24490	3.83259	0.54751	−15.059	48	0.000**
**Pair 2**						
Group 2 pre-test – Group 2 post-test	−1.16667	6.48874	0.88301	−1.321	53	0.192
**Pair 3**						
Group 1 pre-test – Group 2 pre-test	0.95918	15.44474	2.20639	0.435	48	0.666
**Pair 4**						
Group 1 post-test – Group 4 post-test	6.73469	14.44930	2.06419	3.263	48	0.002**

*Source:* Survey data (2019)

* Significant at 5% level;

** significant at 1% level.

From the results in Table 4, a paired sample *t*-test on pair 2 suggests that there was no difference established between before and after values in the control group (*t* [53] = –1.321, *p* = 0.192 [ns]), but a test on pair 1 reveals that there is significant difference (*t* [48] = –15.059, *p* < 0.001) between the pre-test and post-test score of the experiment group, which means a significant impact of treatment was established on the experimental group. Equally, test 4 further confirms that there is significant difference at 1% significant level between the experiment group post-test (group 1) and control group post-test (group 4) (*t* [48] = 3.263, *p* = 0.002).

In addition, pair 3 suggests that the randomisation process was successfully applied to get samples for the experimental and control groups. This was implied by the fact that there was no significant difference (*t* [48] = 435, *p* = 0.666 [ns]) established between the experimental group pre-test (group 1) and control group pre-test (group 2). Hence, assuming that pre-testing has no effect on post-test results, it can be taken that the use of behaviour modification strategies is effective in improving English language reading skills amongst primary school learners. However, there may be some kind of effect of pre-testing on post-test scores because the mean difference increased from –8.245 to 6.735 from pair 1 to 4. This was confirmed through the use of solution with the four control group design, whose results are shown in Table 5.

**TABLE 5:** Paired samples test: Solution with the four control group design.

Pair	Paired differences			*T*	*df*	Sig.
	Mean	Std. deviation	Std. error mean			
**Pair 1**						
Group 1 pre-test – Group 1 post-test	−8.24490	3.83259	0.54751	−15.059	48	0.000
**Pair 2**						
Group 2 pre-test – Group 2 post-test	−1.16667	6.48874	0.88301	−1.321	53	0.192
**Pair 3**						
Group 1 pre-test – Group 2 pre-test	0.95918	15.44474	2.20639	0.435	48	0.666
**Pair 4**						
Group 1 pre-test – Group 2 post-test	−0.42857	16.86589	2.40941	−0.178	48	0.860
**Pair 5**						
Group 3 post-test – Group 4 post-test	5.97959	12.17321	1.73903	3.438	48	0.001
**Pair 6**						
Group 2 pre-test – Group 3 post-test	−8.44898	14.24205	2.03458	−4.153	48	0.000
**Pair 7**						
Group 1 post-test – Group 3 post-test	0.75510	9.54928	1.36418	0.554	48	0.582
**Pair 8**						
Group 2 post-test – Group 4 post-test	−1.03774	17.63515	2.42237	−0.428	52	0.670

*Source:* Survey data (2019)

From Table 5, a paired sample test for Pair 2 suggests that there was no statistically significant difference in reading ability found between before and after values in the control group, control group pre-test (group 2) and control group post-test (group 2) (*t* [53] = –1.321, *p* = 0.192 [ns]). On the contrary, test results for pair 1 reveal that there is statistically significant difference at 1% significance level between pre-test and post-test scores of the experiment group 1 (*t* [48] = –15.059, *p* < 0.01)], implying that a significant effect was found in the use of behaviour modification strategies in improving learner English language reading skills. Furthermore, from the test in pair 3 it was concluded that the randomisation process was effective during sampling of the experiment and control groups because no significant difference was found between control group pre-test and experimental group pre-test (*p* = 0.666).

### Regression analysis model summary on selected behaviour modification practices and English language reading abilities

The study also examined the effect of selected behaviour modification practices to enhance reinforcement of reading abilities amongst dyslexic learners. This was done by the use of multiple regression analysis, where all four behaviour modification practices were factored in the model. The multiple regression also provided information about the relative contribution of each of the variables that make up the model. Each aspect of behaviour modification practices was evaluated in terms of its predictive power, over and above that offered by all the other behaviour modification practices. It enabled the researcher to know how much unique variance, in the dependent variable, each of the independent variables explained. This is shown by coefficients values in Table 6.

**TABLE 6:** Regression analysis model summary output: Behaviour modification practices and English language reading abilities.

Model	*R*	*R*^2^	Adjusted *R*^2^	Std. error of the estimate	Change statistics				
					*R*^2^ change	*F* change	*df*1	*df*2	Sig. *F* change
1	0.330†	0.109	0.104	10.004	0.109	24.603	1	202	0.000
2	0.740‡	0.547	0.536	7.203	0.439	47.927	4	198	0.000

*Source:* Survey data (2019)

† , Predictors: (Constant), group.

‡ , Predictors: (Constant), group, modelling, prompting, shaping, coaching.

¶ , Dependent variable: Post-test.

In Table 6, the variable for block 1 is group of the respondents (experimental or control), which was controlled for in the analysis, while block 2 represents the predictor variables (modelling, prompting, shaping and coaching) together with the control variable (respondent group). It is evident that the respondent’s group alone accounted for 10.9%, as signified by coefficient of *R*^2^ = 0.109, of the variation in the level of English language reading abilities amongst primary school learners with dyslexia. However, after the aspects of behaviour modification practices were included in block 2, it is clear that the model as a whole explained 54.7% (*R*^2^ = 0.547) of the variability in the level of English language reading abilities amongst primary school learners with dyslexia. *R*-square change (0.439) in block 2 indicates the additional amount of variance accounted for by the behaviour modification practices after that explained by the control variable (respondents group) was removed. This indicates that behaviour modification practices accounted for 54.7% (*R*^2^ = 0.547), translating to addition of 43.9% in the variability of in the level of English language reading abilities amongst learners with dyslexia after the effect of the respondents’ group has been statistically removed.

The study further sought to develop a regression model for the relationship between the level of English language reading abilities and behaviour modification practices amongst learners with dyslexia amongst primary school learners. This model was appropriate because each of the explanatory variables was independent and non-mutually exclusive. Table 7 shows the coefficient values of each aspect of behaviour modification practices:

In this model: *Y* = β_0_ + β_1_*X*_1_ + β_2_*X*_2_ + β_3_*X*_3_ + β_4_*X*_4_ + β_5_*X*_5_ + ε,

where: *Y* is English language reading abilities

*X*_1_: group, *X*_2_: prompting, *X*_3_: shaping, *X*_4_: coaching, *X*_5_: modelling.

**TABLE 7:** Coefficient output: Behaviour modification practices and English language reading abilities.

Model	Unstandardised coefficients		Standardised coefficients	*t*	Sig.
	*B*	Std. error	Beta		
**1**					
(Constant)	39.719	2.243	-	17.711	0.000
Group	−6.954	1.402	−0.330	−4.960	0.000
**2**					
(Constant)	0.154	5.200	-	0.030	0.976
Group	−1.927	1.077	−0.091	−1.789	0.075
Prompting	0.512	1.333	0.026	0.385	0.701
Shaping	0.294	1.397	0.01518	0.103	0.834
Coaching	10.774	1.178	0.737	9.147	0.000
Modelling	1.384	1.353	0.066	0.308	1.723

*Source:* Survey data (2019)

† , Dependent variable: post-test.

The predicated optimum level of English language reading abilities was presented by:

0.154 units – 1.927 *X*_1_ units + 0.512 *X*_2_ units + 0.294 *X*_3_ units + 10.774 *X*_4_ units + 1.384 *X*_5_ units + error.

From the model presented in Table 7, the coefficients indicate how much English language reading abilities amongst primary school learners with dyslexia varies with each practice when other behaviour modification practices are held constant. A learner who was treated on behaviour modification practices would perform better in English language reading abilities than the ones who were in the control group by 1.927 units. It emerged that coaching behaviour modification practice had the highest influence on English language reading abilities. This was reflected by the unstandardised coefficient, *X*_4_, which is equal to 10.774, meaning that for each one unit increase in coaching behaviour modification practice, there is an increase in English language reading abilities amongst the primary school learners of 10.774 units. On the contrary, shaping behaviour modification practice reflected the least effect on English language reading abilities. A unit increase in perceived behaviour control would only result in 0.294 units’ improvement in English language reading abilities amongst primary school learners. However, in general, it was concluded that the model was adequate to predict English language reading abilities amongst primary school learners as it accounted for a fairly large variability of 54.7% (*R*^2^ = 0.547).

## Discussion

The study investigated using behaviour modification practices to enhance reinforcement of reading abilities amongst dyslexic learners in Kenya. The findings indicated difference between pre-test and post-test scores of the Experiment group, implying that a significant effect was found in the use of behaviour modification strategies in improving learner English language reading skills. It was also concluded that the randomisation process was effective during sampling of the experiment and control groups because no significant difference was found between control group pre-test and experimental group pre-test. It was therefore concluded that the use of selected behaviour modification practices was effective in improving dyslexic learners’ abilities in the reading of English language. This finding agrees with Altin, Saracaloglu and Boylan (2018), who established that reading comprehension instruction supplemented with traditional materials positively changed learners’ vocabulary knowledge and attitudes towards English lessons. Similarly, Kraft, Blazar and Hogan (2016) agreed that coaching led to improved instruction and achievement. Hayes (2010) also concurred that prompting was effective in teaching autistic children. Mims (2009) also reported that students increased the number of correctly answered comprehension questions during all three shared stories. Aurah (2018) also agreed that use of metacognitive prompts had more positive effects than the conventional method of testing. This finding is further supported by Skinner’s (2011) theoretical assertion that when a behaviour is strengthened it increases the chances and speed of acquisition of a new behaviour. In agreement, Ooko et al. (2019) also indicated that there was a statistically significant positive relationship between shaping and reading abilities. On the contrary, Choi et al. (2016) established that there was no statistically significant difference in reading score growth between experimental and comparison groups. Similarly, Bridge (2016) found that there was no significant difference in development of reading comprehension when two instruction methods were used. The implication of this finding is that teachers need to be trained on behaviour modification practices for enhancement of reading abilities amongst dyslexic learners.

The study reported that coaching behaviour modification practice had the highest influence on English language reading abilities. However, shaping behaviour modification practice reflected the least effect on English language reading abilities. Similarly, Zoccolotti et al. (2014) agreed that orthographic decoding and integration of reading sub-components contributed significantly to the overall prediction of text reading fluency. Ambrose and Cheong (2011) also reiterate that the Clay Modeling Program improves the reading behaviour of dyslexic children. Davis et al. (2018) in the United States of America also agreed that coaching increased student reading achievements. Furthermore, Altin et al. (2018) contend that reading comprehension instruction supplemented with traditional materials positively changed learners’ vocabulary knowledge and attitudes towards English lessons. In addition, Mosito et al. (2015) found a positive significant correlation between use of prompts and reading ability. On the contrary, Loh (2009) disagreed that teachers do not coach reading. The finding that the use of the selected behaviour modification practices was effective in enhancing reading abilities amongst dyslexic learners agrees with Skinner’s theoretical assertion that positive reinforcement is more useful in modifying and reinforcing an already existing behaviour. Therefore, the four selected intervention techniques, namely, prompting, shaping, coaching and modelling, are effective positive reinforcers. The implication of this finding is that the Ministry of Education should train coaching behaviour modification to teachers in schools. The Ministry of Education should set up a learning support area in schools to help manage those learners with dyslexic conditions. It should also equip the learning support departments in the public primary schools with the latest strategies for handling dyslexia in classes.

## Conclusion

The study concludes that the behaviour modification practices model was adequate to predict English language reading abilities amongst primary school learners. The results were statistically significant and accounted for a fairly large variability in English language reading abilities amongst primary school learners. Therefore, implementation of intervention using behaviour modification practices could enhance English language reading abilities of dyslexic primary school learners. It emerged that when other behaviour modification practices were held constant, coaching had the highest influence in English reading ability, followed by prompting and then modelling, while shaping behaviour modification was the least influential. It can be concluded that a learner treated on behaviour modification practices would perform better in English language reading ability than the ones not given the intervention. Therefore, teachers in schools should be sensitised on handling dyslexic learners in their classes to offer appropriate intervention measures to help the dyslexic learners. Moreover, teacher counsellors should include the parents of the dyslexic learners in strategising how to improve their social competencies and improve the welfare both in and out of school. One of the limitations that arose from the experiment is that there are differences amongst the public schools where the research was conducted and this could have brought slight differences in the efficacy of the behaviour modification intervention. However, this was addressed because there was known structured intervention literacy being practised in the selected schools; hence, the results could still be treated as accurate and generalisable. Future studies could investigate the effectiveness of behaviour modification practices with longer time interventions. In addition, gender differences in the response to behaviour modification interventions could be explored.
